# Combination of IVIM-DWI and 3D-ASL for differentiating true progression from pseudoprogression of Glioblastoma multiforme after concurrent chemoradiotherapy: study protocol of a prospective diagnostic trial

**DOI:** 10.1186/s12880-017-0183-y

**Published:** 2017-02-01

**Authors:** Zhi-Cheng Liu, Lin-Feng Yan, Yu-Chuan Hu, Ying-Zhi Sun, Qiang Tian, Hai-Yan Nan, Ying Yu, Qian Sun, Wen Wang, Guang-Bin Cui

**Affiliations:** Department of Radiology, Tangdu Hospital, Fourth Military Medical University, 569 Xinsi Road, Xi’an, 710038 China

**Keywords:** Glioblastoma multiforme, True progression, Pseudoprogression, Intravoxel incoherent motion diffusion-weighted imaging, Diffusion weighted imaging, Perfusion, 3D arterial spin labeling

## Abstract

**Background:**

Standard therapy for Glioblastoma multiforme (GBM) involves maximal safe tumor resection followed with radiotherapy and concurrent adjuvant temozolomide. About 20 to 30% patients undergoing their first post-radiation MRI show increased contrast enhancement which eventually recovers without any new treatment. This phenomenon is referred to as pseudoprogression. Differentiating tumor progression from pseudoprogression is critical for determining tumor treatment, yet this capacity remains a challenge for conventional magnetic resonance imaging (MRI). Thus, a prospective diagnostic trial has been established that utilizes multimodal MRI techniques to detect tumor progression at its early stage. The purpose of this trial is to explore the potential role of intravoxel incoherent motion diffusion-weighted imaging (IVIM-DWI) and three-dimensional arterial spin labeling imaging (3D-ASL) in differentiating true progression from pseudoprogression of GBM. In addition, the diagnostic performance of quantitative parameters obtained from IVIM-DWI and 3D-ASL, including apparent diffusion coefficient (ADC), slow diffusion coefficient (D), fast diffusion coefficient (D*), perfusion fraction (f), and cerebral blood flow (CBF), will be evaluated.

**Methods:**

Patients that recently received a histopathological diagnosis of GBM at our hospital are eligible for enrollment. The patients selected will receive standard concurrent chemoradiotherapy and adjuvant temozolomide after surgery, and then will undergo conventional MRI, IVIM-DWI, 3D-ASL, and contrast-enhanced MRI. The quantitative parameters, ADC, *D*, D*, *f*, and CBF, will be estimated for newly developed enhanced lesions. Further comparisons will be made with unpaired *t*-tests to evaluate parameter performance in differentiating true progression from pseudoprogression, while receiver-operating characteristic (ROC) analyses will determine the optimal thresholds, as well as sensitivity and specificity. Finally, relationships between these parameters will be assessed with Pearson’s correlation and partial correlation analyses.

**Discussion:**

The results of this study may demonstrate the potential value of using multimodal MRI techniques to differentiate true progression from pseudoprogression in its early stages to help decision making in early intervention and improve the prognosis of GBM.

**Trial registration:**

This study has been registered at ClinicalTrials.gov (NCT02622620) on November 18, 2015 and published on March 28, 2016.

## Highlights

- Intravoxel incoherent motion (IVIM) magnetic resonance imaging (MRI) allows the simultaneous acquisition of diffusion and perfusion parameters which reflect tumor cellularity and vascularity, respectively.
- Arterial spin labeling (ASL) and IVIM are two commonly used perfusion MRI techniques without introducing contrast agents and are considered as safe and reliable methods.

## Background

Glioblastoma multiforme (GBM) is the most common malignant primary brain tumor in adults. A combination of radiation therapy with concurrent or adjuvant temozolomide (TMZ) following maximum safe tumor resection can significantly improve patients’ survival, and is currently the standard treatment for GBM. Even with this standard treatment, the survival of GBM patients remains extremely poor and the overall median survival time is 14–18 months after treatment [1, 2].

GBM treatment efficacy is generally evaluated with contrast-enhanced MRI in conjunction with clinical assessment. Only recently have radiologists and clinicians observed some transient treatment-induced changes after radiotherapy which demonstrate tumor progression features in contrast-enhanced MRI such as progressive enlargement and new enhancement. However, these features mainly derive from postsurgical changes including radiation effects, treatment-induced inflammation, and ischemia [3, 4]. This phenomenon is referred to as pseudoprogression. Chemotherapy with TMZ significantly increased the rate of pseudoprogression [5, 6]. It is reported that half of the patients with high-grade gliomas after chemoradiotherapy showed an enlarged enhancement area on contrast-enhanced T1-weighted magnetic resonance (MR) images, of which 20 to 30% were pseudoprogression [7–9].

While both tumor progression and pseudoprogression exhibit progressive enlargement and new enhancement within a radiation field [3], the treatment and prognosis for them are totally different. Tumor progression reflects treatment failure and the treatment plan should be adjusted accordingly [10], while pseudoprogression is associated with a favorable prognosis and can improve spontaneously with adjuvant TMZ. If the latter is misdiagnosed as true progression, the treatment efficacy can be underestimated, thus leading to the inappropriate adjust of therapy. However, true progression can not be easily differentiated from pseudoprogression with current conventional MRI sequences. Therefore, new imaging tools that can assist clinicians in effectively identifying true progression versus pseudoprogression within 6 months after concurrent chemoradiotherapy are needed, which will facilitate the selection of appropriate treatment or the early termination of an invalid treatment plan.

There have been several studies in which researchers differentiated true progression from treatment effects by using advanced MR imaging techniques, such as diffusion weighted magnetic resonance imaging (DWI) and dynamic susceptibility-weighted contrast-enhanced (DSC) MRI. True progression exhibits significantly lower apparent diffusion coefficient (ADC) values compared with pseudoprogression [11, 12], reflecting the high cellularity of progressed tumor. Considering that ADC values can be compromised by perfusion, Le Bihan et al. introduced an intravoxel incoherent motion (IVIM) technique [13, 14]. Slow diffusion coefficient (D) is the diffusion parameter which reflects the diffusion coefficient for water while fast diffusion coefficient (D*) represents perfusion-related diffusion. Fraction of fast ADC (f) is the perfusion fraction linked to microcirculation which has been successfully applied to glioma grading and to differentiate recurrent tumors from treatment-related changes [15, 16]. DSC is a T2*-weighted technique to measure relative cerebral blood volume (rCBV) and lower mean rCBV values in pseudoprogression was previously used to differentiate from true progression [17]. However, radiotherapy and chemotherapy for GBM destroy blood–brain barrier, allowing gadolinium-based contrast agents to leak into the interstitial fluid, which may lead to the underestimation of rCBV with DSC [18]. Three-dimensional arterial spin labeling (3D-ASL) is another non-invasive and contrast agent-free perfusion imaging method to measure cerebral blood flow (CBF) [19]. The diagnostic performance of 3D-ASL has been found to be equivalent to DSC MRI in assessing brain tumor perfusion, particularly for those patients not suitable for DSC because of renal failure [20].

Despite the application of IVIM and 3D-ASL in glioma study, there still lacks study in which IVIM is combined with 3D-ASL in the same GBM patient cohort to evaluate their efficacy of differentiate true tumor progression from pseudoprogression as a consequnence of radiochemotherapy for GBM.

Therefore, the current prospective diagnostic trial was designed to evaluate the ability of IVIM-DWI and 3D-ASL to distinguish true tumor progression from pseudoprogression in GBM. The diagnostic performance of the quantitative parameters obtained from IVIM-DWI and 3D-ASL will also be compared.

## Methods/Design

**Fig. 1:**
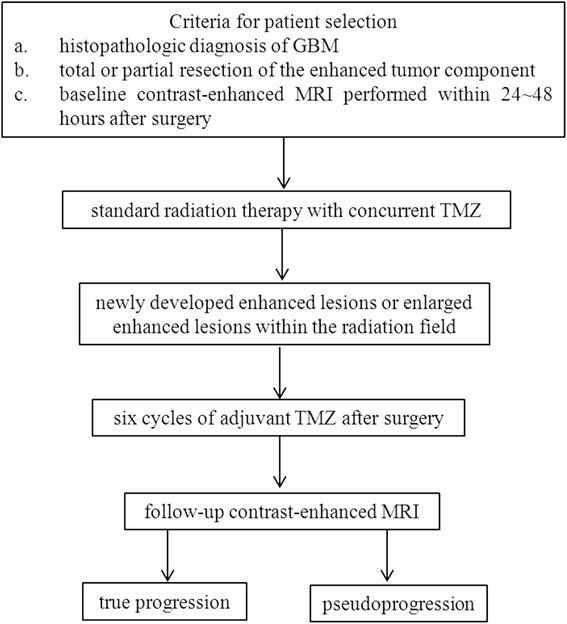
Flowchart of the current prospective diagnostic trial

This prospective single institution observational study will include two case groups and has been approved by the Ethics Committee of Tangdu Hospital (TDLL-20151013). The scheme of the current trial is presented in Fig. 1.

### Patient Selection

#### Inclusion criteria

Patients that recently received a histopathological diagnosis of GBM from Tangdu Hospital are considered for enrollment in the present study. The criteria for selection are: (1) a recent histopathologic diagnosis of GBM according to criteria of the World Health Organization; (2) total or partial resection of the enhanced tumor component; (3) baseline contrast-enhanced MRI performed within 24 ~ 48 h after surgery; (4) standard radiation therapy with concurrent TMZ and six cycles of adjuvant TMZ after surgery; (5) follow-up IVIM-DWI and 3D-ASL performed on the same 3 T MRI scanner within 6 months after the completion of radiation therapy with concurrent TMZ, when pseudoprogression is prevalent [21], however as a long project, we will follow up glioma patients as long as possible;(6) Without receiving corticosteroid management at 3 days before imaging;(7) the presence of newly developed enhanced lesions or enlarged enhanced lesions within the radiation field; (8) surgical resection of enhanced tissue or adequate clinicoradiologic follow-up to definitively diagnose true progression or pseudoprogression according to Response Assessment in Neuro-Oncology (RANO) criteria [22].

#### Exclusion criteria

Subjects will be excluded based on any of the following conditions: (1) without contrast-enhanced MRI performed within 24 ~ 48 h after surgery; (2) absence of newly developed enhanced lesions or enlarged enhanced lesions after the end of radiation therapy with concurrent TMZ; (3) non-standard treatment after surgery; or (4) patient does not complete clinicoradiologic follow-up or surgical resection of enhanced tissue to definitively diagnose true progression or pseudoprogression.

### IVIM model

DWI dada will be analyzed using the same protocol as described in our previous publication [15]. DWI will be analyzed with an IVIM model (Eq. 1), where *S0* and *S*(*b*) are the signal intensities of attenuation at a *b-*value of 0 s/mm^2^ and at a *b*-value > 0 s/mm^2^, respectively. *D* is the diffusion parameter which reflects the diffusion coefficient for water, D* represents perfusion-related diffusion and *f* is the perfusion fraction linked to microcirculation.

Considering that D* is significantly larger than D [14, 23]. The contribution of D* to signal decay can be neglected with a *b*-value > 200 s/mm^2^. Thus, Eq. (1) can then be simplified, and the estimation of D can be obtained by using only b values larger than 200 s/mm^2^, with a simple linear fit Eq. (2), then D* and *f* will be generated with low *b*-values by using a nonlinear regression algorithm based on Eq. (1).

1 $$ S(b)/ S 0= f\; exp\left(- b\times D*\right) + \left(1- f\right)\  exp\ \left(- b\times D\right) $$

2 $$ S(b)\ / S 0 = exp\ \left(- bD\right) $$

The ADC value will be derived from a monoexponential equation with all *b*-values by Eq. (3).

3 $$ S(b)\ / S 0 = exp - \left( ADC \times b\right) $$

### Image acquisition

Upon completion of radiation therapy with concurrent TMZ, follow-up MRI scans will be performed with a 3.0 T MRI system (MR750; GE Healthcare, Milwaukee, WI, USA) with an 8-channel head coil. Conventional MRI, DWI with 13 b-values (0 ~ 1500 s/mm^2^), 3D-ASL, and contrast-enhanced MRI will be arranged in regular sequence for each patient’s first follow-up visit. Conventional MRI sequences will include spin-echo T1-weighted imaging, fast spin-echo T2-weighted imaging, and fluid-attenuated inversion recovery imaging (FLAIR). The IVIM DWI will be performed prior to gadolinium injection with a single-shot diffusion-weighted spin-echo echo-planar sequence using 13 different b-values: 0, 10, 20, 30, 50, 80, 100, 200, 300, 500, 800, 1000, and 1500s/mm^2^. These b-values were selected to cover both initial pseudo diffusion decay (b < 200 s/mm^2^) and molecular diffusion decay (b ≥ 200 s/mm^2^) [24]. In total, 20 axial slices covering the entire brain will be obtained. The following MR imaging parameters will be used: field of view (FOV) = 256 × 256 mm^2^, slice thickness = 5 mm, slice gap = 1.5 mm, repetition time (TR) = 3000 ms, echo time (TE) = minimum, matrix =128 × 128. The total acquisition time will be 5 min and 45 s.

A 3D spiral fast spin echo (FSE) sequence will be used to obtain 3D-ASL perfusion images. The MRI parameters will be: 512 sampling points on eight spiral arms, spatial resolution = 3.64 mm, TR = 4590 ms, TE = 10.5 ms, slice thickness = 4.0 mm, number of slices = 40, FOV = 240 × 240 mm^2^, number of excitations (NEX) = 3.0. The total acquisition time will be 4 min.

Finally, a contrast-enhanced T1-weighted spin echo sequence will be performed in the transverse, sagittal, and coronal planes following a bolus injection of 0.1 mmol/kg of gadodiamide (Omniscan; GE Healthcare, Co. Cork, Ireland).

### Image analysis

All imaging data will be transferred to a GE ADW4.6 workstation. All of the IVIM-DWI and 3D-ASL parameters will be measured by two experienced neuroradiologist in consensus (SYZ, TQ, with 9 and 8 years of clinical experience in neuroradiology), who are blinded to the clinical outcome. The conventional plain, contrast-enhanced MRI scans will be carefully reviewed to detect if there are newly developed enhanced lesions within the radiation field. The section containing the maximum diameter of the enhanced lesion will be selected for subsequent regions of interest (ROI) analysis. ROI will be manually extracted to cover as much of the solid parts of the enhanced lesions as possible on a single section, while avoiding large vessels and hemorrhagic, ischemic, cystic, and necrotic areas according to the anatomical contrast images. The ROI will be transferred to the IVIM parametric maps. Diffusion and perfusion parameter maps of the IVIM will be generated automatically and the parameters will be obtained from the ROI, including mean ADC, *D*, D*, and *f*.

ROI analysis for 3D-ASL will be performed in a manner similar to that of IVIM-DWI. A single contrast section containing the maximum diameter of the enhanced lesion will be selected, and an ROI will be drawn around the entire enhanced lesion, while avoiding large vessels and hemorrhagic, ischemic, cystic, and necrotic areas according to the anatomical contrast images. The ROI will be transferred to the 3D-ASL parametric maps. the mean CBF parameters will be generated automatically.

### Follow-up and lesion diagnosis

Serial follow-up MRI assessments will be performed approximately 2 months after the completion of standard radiation therapy with concurrent TMZ. Disease progression will be definited according to RANO criteria or histopathology of surgical resection. If the size of the enhanced lesions remain unchanged or show a decrease for at least 6 months, pseudoprogression will be defined. On the contrary, if the enhanced lesions exhibit a gradual increase in size for at least 6 months, true progression will be defined. Radiological evaluation will be made by two authors in consensus (SYZ, TQ with 9 and 8 years of clinical experience in neuroradiology).. Pseudoprogression will be defined as some treatment effects with the complete absence of tumor. Recurrent tumor will be defined as any amount of tumor. Pathologic evaluation will be made by an experienced neuropathologist (WZ, with 26 years of clinical experience in neuropathology).

### Sample size calculation and statistics

A sample size calculation was performed with reference to a previous study that had a sensitivity of 71.0% and a specificity of 75.0% [16]. With a permissible error of 0.1 and an alpha significance level of 0.05, a sample size of 50 patients per group was enough to get satisfying statistic power.

Numerical variables will be expressed as the mean ± standard deviation (SD). Student’s *t*-test will be used to assess significant differences in the above mentioned parameters between the true tumor progression and the pseudoprogression cases. Receiver operating characteristic (ROC) analyses will determine corresponding specificity, sensitivity and cutoff points for differentiating true tumor progression from pseudoprogression based on ADC, *D*, D*, *f*, and CBF values. Associations between *f*, *D*, and corresponding CBF and ADC will be assessed with Pearson’s correlation and partial correlation analyses, respectively. SPSS 17.0 software (SPSS Inc, Chicago, IL, USA) will be used for all statistical analyses and *p* < 0.05 indicate statistical significance.

### Primary outcome measure

1. Differences in diffusion-related parameters between true tumor progression and pseudoprogression according to the different fitting model
*D* and ADC will be derived from biexponential and monoexponential models, respectively. We hypothesized that true tumor progression and pseudoprogression can be distinguished based on these two values and true tumor progression can exhibit significantly lower mean *D* and ADC values compared with pseudoprogression. In addition, we hypothesized that *D*, which separates perfusion effects, will be a more promising parameter in distinguishing true tumor progression from pseudoprogression compared with ADC which can be compromised by perfusion.
2. Differences in perfusion-related parameters between true progression and pseudoprogression
  Data regarding D*, *f*, and CBF will be summarized. We hypothesized that D*, *f*, and CBF will significantly differ between true tumor progression and pseudoprogression and Mean D*, f, and CBF values will be signifcantly lower for pseudoprogression than for true progression.
3. Diagnostic Performance of the IVIM and 3D-ASL Parameters
  Receiver operating characteristic (ROC) analyses will determine corresponding cutoff points for differentiating true tumor progression from pseudoprogression based on ADC, *D*, D*, *f*, and CBF values. In addition, sensitivity, specificity, and area under curve (AUC) for identifying true progression will be calculated in each case when pseudoprogression is differentiated. We will make a comparison among all the IVIM and 3D-ASL parameters to identify which has the best sensitivity/specificity with regards to early diagnosis.
4. Establishment of Clinical prediction model
  Single factor analysis will show which covariates affect the judgment of newly developed enhanced or enlarged enhanced lesions, such as age, gender, radiation dose, karnofsky performance score, lesion region and imaging characteristics. A logistic regression equation will be performed to identify those covariates, IVIM and 3D-ASL parameters that contribute to the diagnostic differentiation between true progression and pseudoprogression.

### Secondary outcome measures

1. Correlations among imaging parameters
  Correlation coefficients between *f* and corresponding CBF values, and between *D* derived from the biexponential model and corresponding ADC values derived from the monoexponential model, will be calculated. It is hypothesized that both sets of correlations will be significant.

## Discussion

In patients with GBM after concurrent chemoradiotherapy, it is important to differentiate pseudoprogression from true progression to choose appropriate treatment and predict prognosis. In both situations, conventional MRI usually shows enhanced lesion with or without mass effect, so it cannot be conclusive. Few studies have examined the combination of IVIM and 3D-ASL imaging for distinguishing true progression from pseudoprogression of GBM, The current study attempts to validate perfusion and diffusion parameters derived from IVIM and 3D-ASL to determine whether an enlarged contrast-enhanced lesion was caused by true progression or by pseudoprogression. Advantages and disadvantages of this study are discussed below.

Compared to recently published studies in gliomas [15, 25], more and higher b values are used in our study, as more b values in segment of low b values can improve the accuracy of the pseudodiffusion, while higher b values can better eliminate the perfusion-related diffusion; thus it can in turn generate a more realistic molecular diffusion coefficient value. Perfusion imaging of brain tumors, which mainly includes DSC-MR perfusion techniques, has been used for the diagnosis of glioma recurrence and radiation necrosis [26–28]. However, the use of intravenous contrast media is a major limitation in the routine clinical application of this method, since contrast media extravasation can result in a decreased rCBV for high-grade tumors. The 3D-ASL is one submethod of ASL. It had been widely used in gliomas grading and had reliable results with DSC. In this technique, the contrast agent used is labeled arterial blood water proximal to the brain. Without intravenous contrast media, 3D-ASL imaging could be particularly relevant for the long-term follow-up of gliomas following radiation, including those with renal insufficiency or severe allergies. Several reports [26–28] have suggested that combining information derived from different techniques increases diagnostic performance not only for the identifcation of brain tumors but also for assessing the response to treatment. Because each parameter can show different aspects of tumor biology, the combination of parameters may have added value compared with single-parameter measurements.

We are expecting that combination of IVIM-DWI and 3D-ASL imaging has an acceptable accuracy in differentiating true progression from pseudoprogression However, there are several limitations of our study. First, patient recruiting is difficult. Second, manual ROI measurements that encompassed the entire lesion and targeted the areas of maximal abnormality is used to measure the parameters rather than a pixel-to-pixel method. This method may not reflect the intraregional tumor heterogeneity. However, these techniques are rapid, reproducible, simple, and are commonly used in clinical practice. Third, pathologic sampling regions lacks strict pathologic correlation of the image-based segmentation with surgical specimens, as shown by Hu et al. [10]. However, in clinical practice, such quantitative correlation is very difficult to achieve.

## Abbreviations

3D-ASL: Three-dimensional arterial spin labeling imaging
ADC: Apparent diffusion coefficient
AUC: Area under curve
CBF: Cerebral blood flow
*D**: Fast diffusion coefficient
*D*: Slow diffusion coefficient
DSC: Dynamic susceptibility-weighted contrast-enhanced
*f*: Perfusion fraction
FA: Fractional anisotropy
FOV: Field of view
FSE: Fast spin echo
GBM: Glioblastoma multiforme
IVIM-DWI: Intravoxel incoherent motion diffusion-weighted imaging
MRI: Magnetic resonance imaging
NEX: Number of excitations
ROC: Receiver-operating characteristic
TMZ: Temozolomide
SD: Standard deviation
TE: Echo time
TR: Repetition time
